# Disruption of SHH signaling cascade by SBE attenuates lung cancer progression and sensitizes DDP treatment

**DOI:** 10.1038/s41598-017-02063-x

**Published:** 2017-05-15

**Authors:** Jing Du, Weiwei Chen, Lijuan Yang, Juanjuan Dai, Jiwei Guo, Yan Wu, Kaikai Gong, Jian Zhang, Ning Yu, Zhen Xie, Sichuan Xi

**Affiliations:** 1 Cancer Research Institute, Binzhou Medical University hospital, 256600 Binzhou, P.R. China; 2 Department of Pathology, Binzhou City People’s Hospital, 256610 Binzhou, P.R. China; 3 Department of Pathology, Binzhou Medical University Hospital, 256600 Binzhou, P.R. China; 4 Department of Thoracic Surgery, Binzhou Medical University Hospital, 256600 Binzhou, P.R. China

## Abstract

Deregulated Sonic Hedgehog (SHH) pathway facilitates the initiation, progression, and metastasis of Non-small cell lung cancer (NSCLC), confers drug resistance and renders a therapeutic interference option to lung cancer patients with poor prognosis. In this study, we screened and evaluated the specificity of a Chinese herb *Scutellariabarbata D. Don* extraction (SBE) in repressing SHH signaling pathway to block NSCLC progression. Our study confirmed that aberrant activation of the SHH signal pathway conferred more proliferative and invasive phenotypes to human lung cancer cells. This study revealed that SBE specifically repressed SHH signaling pathway to interfere the SHH-mediated NSCLC progression and metastasis via arresting cell cycle progression. We also found that SBE significantly sensitized lung cancer cells to chemotherapeutic agent DDP via repressing SHH components *in vitro* and *in vivo*. Mechanistic investigations indicated that SBE transcriptionally and specifically downregulated SMO and consequently attenuated the activities of GLI1 and its downstream targets in SHH signaling pathway, which interacted with cell cycle checkpoint enzymes to arrest cell cycle progression and lead to cellular growth inhibition and migration blockade. Collectively, our results suggest SBE as a novel drug candidate for NSCLC which specifically and sensitively targets SHH signaling pathway.

## Introduction

As the leading cause of cancer mortality with the most common incidence, lung cancer is now therapeutically challenged all over the world^1, 2^. The efficacy in treatment of Non-small cell lung cancer (NSCLC), which account for around 85% of all lung cancer cases, has been limited even with combination of multiple targeted therapies^3–5^. Platinum-based chemotherapy has improved NSCLC patients in long-term survival but with short term of low efficacy, high toxicity and nearly unavoidable development of drug resistance^4, 6, 7^. Therefore, searching for new drugs with high efficacy and low toxicity is urgently demanded.

Given the inefficacy in the current therapeutic strategies for lung cancer, the characterization of the key aberrantly deregulated signal pathways in initiating and maintaining the lung cancer development and progression is the first critical step in innovating even revolutionizing our current lung cancer therapeutic choices. The Hedgehog pathway, which plays a principle role in organ development, is recently found to be aberrantly stimulated in various cancers including lung cancer, breast cancer, glioma and basal cell carcinoma^8^. As one of essential signaling cascades characterized well in stem cell differentiation and proliferation, Sonic Hedgehog (SHH) pathway interacts with other cancer-associated signaling molecules like RAS/RAF/MEK/ERK, PI3K/AKT/mTOR, EGFR, and Notch^9^. The transmembrane protein Patched (PTCH) from canonical sonic Hedgehog cascade represses the G-protein coupled receptor like protein Smoothened (SMO) from translocating to the primary cilium. Binding of SHH to its receptor PTCH de-represses SMO to enhance the phosphorylation and translocation of GLI1 zinc finger transcription factors into nucleus, followed by activation of downstream targets including SHH, PTCH, GLI1 and cell cycle regulating proteins^10, 11^. As the key regulators of Hedgehog pathway, SHH, SMO and GLI1 are upregulated in NSCLC tumor tissues compared to normal tissues^12–14^. A comprehensive screening of 60 NSCLC cell lines reveals the constitutive activation of SHH signaling in most of those cell lines^15^. Forced expression of GLI1 enhances NSCLC cell proliferation via upregulating Cyclin D1 and D2^16^. SHH pathway-specific inhibitors have been applied to treat drug resistance in cancer therapies for years^16–20^. Preclinical and clinical trials are also underway to test the efficacy of SHH inhibitors in repression of angiogenesis of breast and colon tumors via potentially targeting the VEGF activation^21^.

Traditional Chinese medicines (TCMs) have been being applied independently or combined with modern medicine to improve symptoms, enhance quality of life, prevent recurrence and metastasis, and prolong survival in cancer patients. *Scutellariabarbata D. Don* extraction (SBE), as one of TCMs, has been being used in China for many years as an efficacious anti-cancer drug^22–25^. SBE is reported recently to induce cancer cell apoptosis by activating the mitochondrion-dependent pathway, to repress tumor angiogenesis via suppression of Hedgehog signaling, and to enhance G1/S arrest in human colon carcinoma cells by modulating a number of signaling pathways associated with the cell cycle^22, 26–28^. However, the underlying mechanisms governing the antitumor role of SBE in lung cancer and cancer metastasis are still not fully explored.

In this study, we will dissect the mechanism underlying the specificity of SBE in repressing SHH signaling pathway to block NSCLC progression and metastasis, as well as validate the efficacy of SBE as a potential therapeutic drug candidate for NSCLC patients.

## Results

### Aberrant activation of SHH in lung tumors from patients associates with adverse prognosis

**Figure 1:**
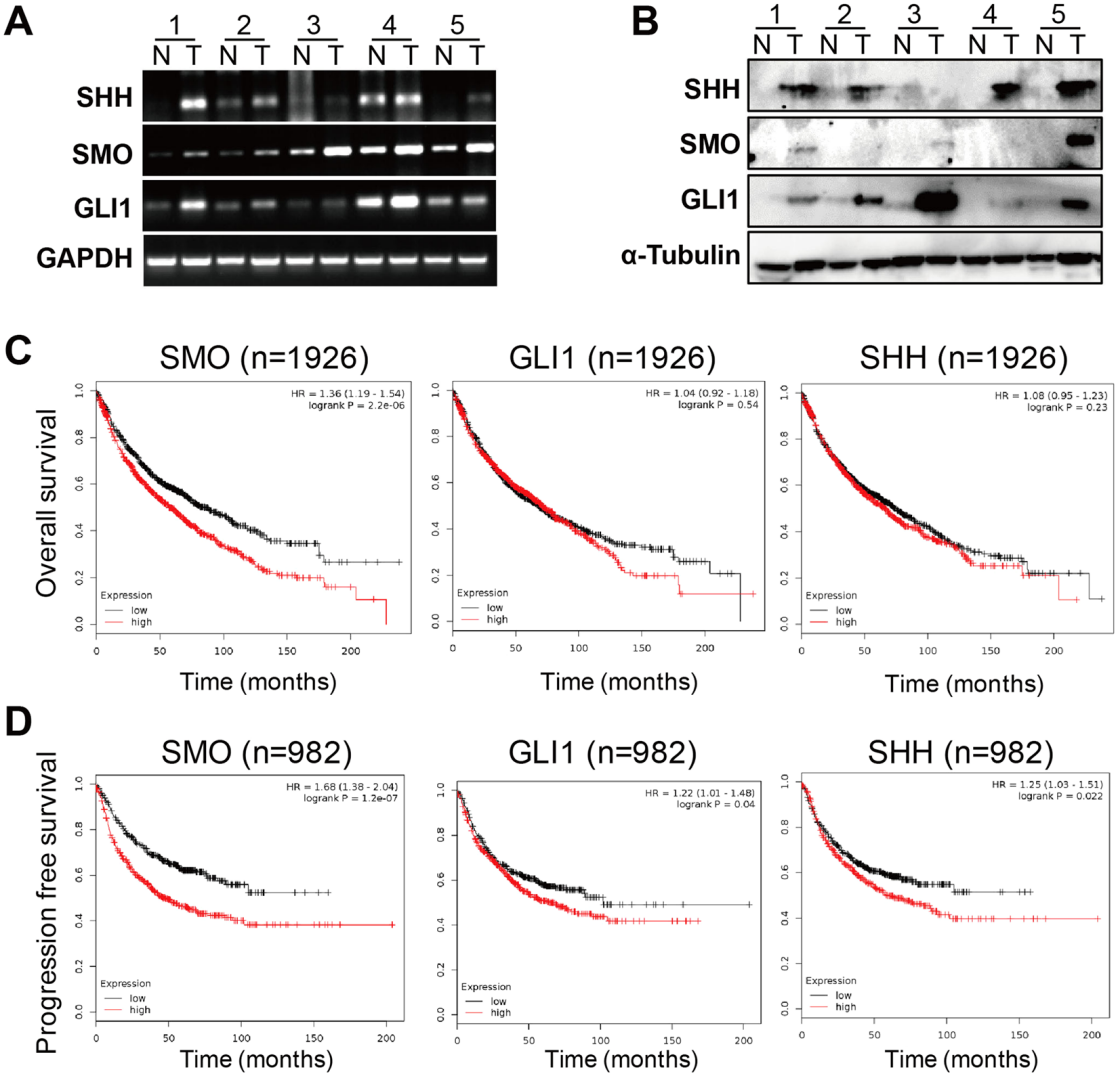
Aberrant activation of SHH signaling in lung tumors from patients with adverse prognosis. (**A**) RT-PCR analysis of the endogenous mRNA levels of SHH, SMO and GLI1 in human lung cancers relative to paired normal lung tissues. GAPDH was amplified in parallel as inner control. N and T represent normal and tumor specimens separately. (**B**) Immunoblotting analysis of the endogenous protein levels of SHH, SMO and GLI1 in human lung cancers relative to paired normal lung tissues. α-Tubulin was loaded as inner control. (**C**) Kaplan Meier overall survival (OS) curves of SMO (left, n = 1926, p = 2.2E-06 by log-rank test for significance), GLI1(middle, n = 1926, p = 0.54 by log-rank test for significance) and SHH (right, n = 1926, p = 0.23 by log-rank test for significance). (**D**) Kaplan Meier progression free survival (PFS) curves of SMO (left, n = 982, p = 1.2E-07 by log-rank test for significance), GLI1(middle, n = 982, p = 0.04 by log-rank test for significance) and SHH (right, n = 982, p = 0.022 by log-rank test for significance).

To examine the expression profile of SHH signaling components for identification of roles of SHH pathway signaling in human lung cancer tissues, we performed both RT-PCR and immunoblotting and found that endogenous mRNA levels of SHH, SMO and GLI1 are significant higher except for SHH in sample #3 in all five human lung cancers relative to paired normal lung tissues (Fig. 1A). As indicated in Fig. 1B, the protein levels of SHH, SMO and GLI1 were also significantly elevated and consistently matched with their mRNA levels in most of those same five lung tumor tissues compared to their adjacent normal lung tissues. The publicly available datasets (2015 version) (http://www.kmplot.com/analysis/index.php?p=service&cancer=lung)^29^ were screened and applied to analyze the prognostic correlation between expression of SMO and GLI1 and survival of lung cancer patients. As the Kaplan-Meier analyses indicated, higher expression level of SMO was highly inversely correlated with shorter overall survival (OS) (n = 1926, p = 2.2 × 10^−6^) (Fig. 1C). A similar anti-correlation was also found between higher level of SMO and shorter progression free survival (PFS) (n = 982, p = 1.2 × 10^−7^) (Fig. 1D). Furthermore, GLI1 was also found to be a negative indicator for PFS (n = 982, p = 0.04) but not OS (n = 1926, p = 0.54) of NSCLC patients (Fig. 1C and D). As a cytokine in the upstream of SHH cascade, SHH transcription level was revealed to be statistically significant relevant to poor outcome with regards to PFS (n = 982, p = 0.022) rather than OS (n = 1926, p = 0.23) (Fig. 1C and D).

### Downregulation of SHH reduces cell proliferation and clonogenicity of NSCLC via cell cycle arrest

**Figure 2:**
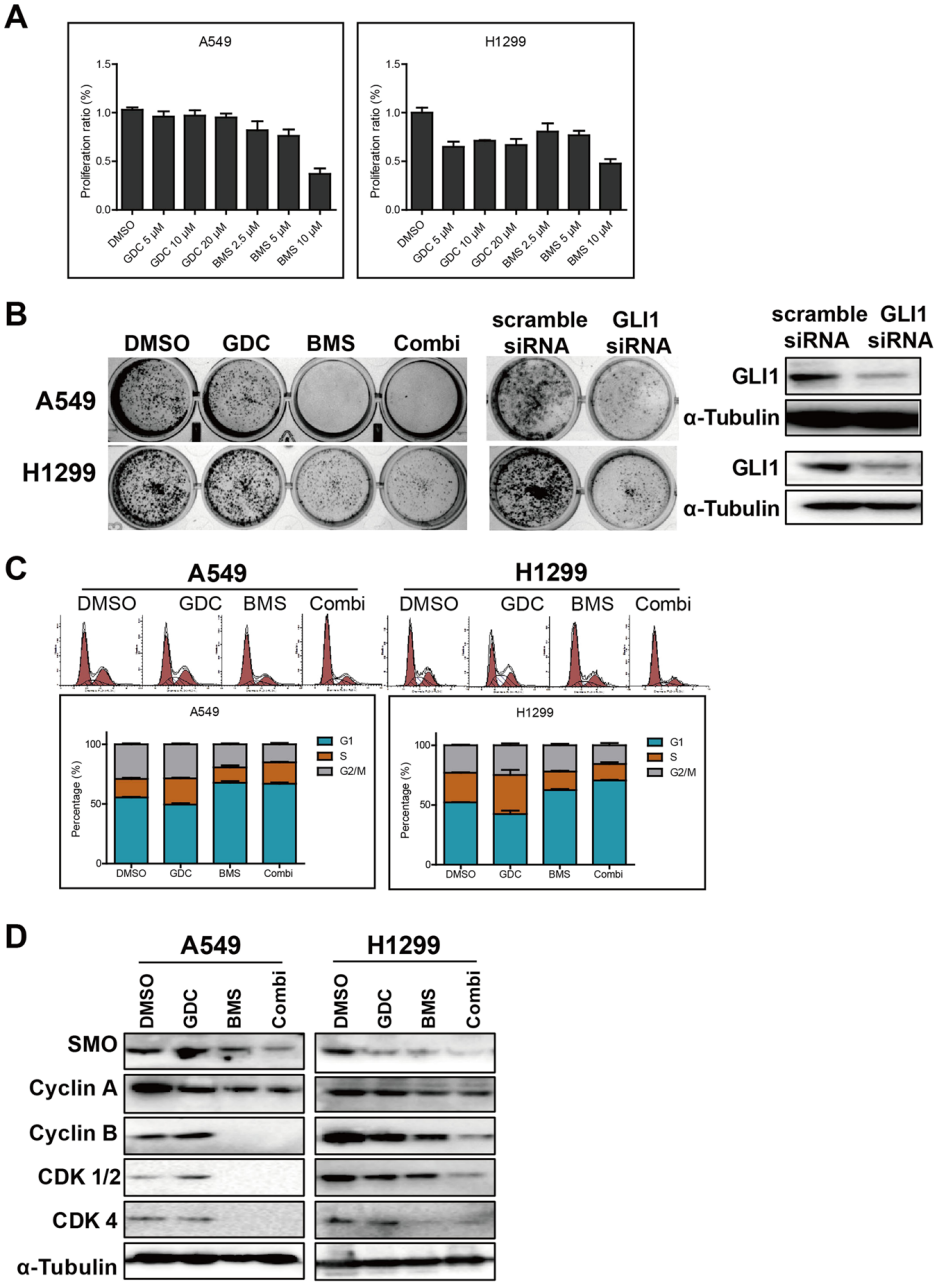
Targeting SHH signaling reduces cell proliferation and clonogenicity of NSCLC via arresting cell cycle. (**A**) CCK-8 assay showing that SMO inhibitors BMS (BMS-833923) inhibited cell proliferation of both A549 and H1299 at the 2.5 µM concentration or higher. SMO inhibitors GDC (GDC-0449) significantly arrested H1299 not A549 cell growth at 5 µM concentration or above. (**B**) The representative images of colony formation of A549 and H1299 cells treated with GDC (10 µΜ), and/or BMS (5 µM), or siRNA-GLI1 from clonogenicity assay. (**C**) Flowcytometry analysis demonstrating that GDC and/or BMS significantly induced G1/S phase arrest in A549 and H1299 cells, in which additive G1/S arrest was induced with co-treatment of GDC and BMS. Data were analyzed by ModFit software and presented as mean ± SD. (**D**) Immunoblotting analysis demonstrating that GDC and/or BMS significantly reduced protein level of SMO and cell cycle regulators (Cyclin A, Cyclin B, CDK1 and CDK4) in A549 and H1299 cells, in which additive repression of SMO and those cell cycle regulators G1/S arrest was detected with co-treatment of GDC and BMS. α-Tubulin was loaded as inner control. All the co-treatments of GDC and BMS were labeled as “Combi” in this figure.

Two SMO inhibitors GDC-0449 (GDC) and BMS-833923 (BMS) for downregulation of SHH signaling was applied to explore whether activation of SHH pathways is involved in growth and clonal expansion of lung cancer cells. The proliferation assay demonstrated relative mild growth inhibition of A549 and H1299 cells after exposure to both GDC and BMS for 48 hours (Fig. 2A). Further clonal formation assay indicated the most dramatic inhibitory effects of SMO inhibitors on clonogenicity of A549 and H1299 cells, in which clonal formation rates were reduced more that 70% for BMS only and more than 90% for BMS plus GDC in those tumor cells with 24 hours’ exposure (Fig. 2B). In addition, specific silencing of GLI1 by siRNA also significantly decreased clonogenicity of A549 and H1299 cells (Fig. 2B). Flow cytometry analysis was performed to search the biological mechanisms underlying the repressed proliferation and clonal expansion. Our data demonstrated that GDC and/or BMS significantly induced G1/S phase arrest in A549 and H1299 cells, in which additive G1/S arrest was induced with co-treatment of GDC and BMS (Fig. 2C). Those cell cycle progression arrests were well explained in following immunoblotting analysis which indicated that GDC and/or BMS significantly reduced protein level of SMO and cell cycle regulators (Cyclin A, Cyclin B, CDK1 and CDK4) in A549 and H1299 cells, in which additive repression of SMO and those cell cycle regulators G1/S arrest was detected with co-treatment of GDC and BMS (Fig. 2D).

### SBE inhibits cell proliferation and clonogenicity of NSCLC via selectively repressing SHH pathway and cell cycle checkpoint enzymes

**Figure 3:**
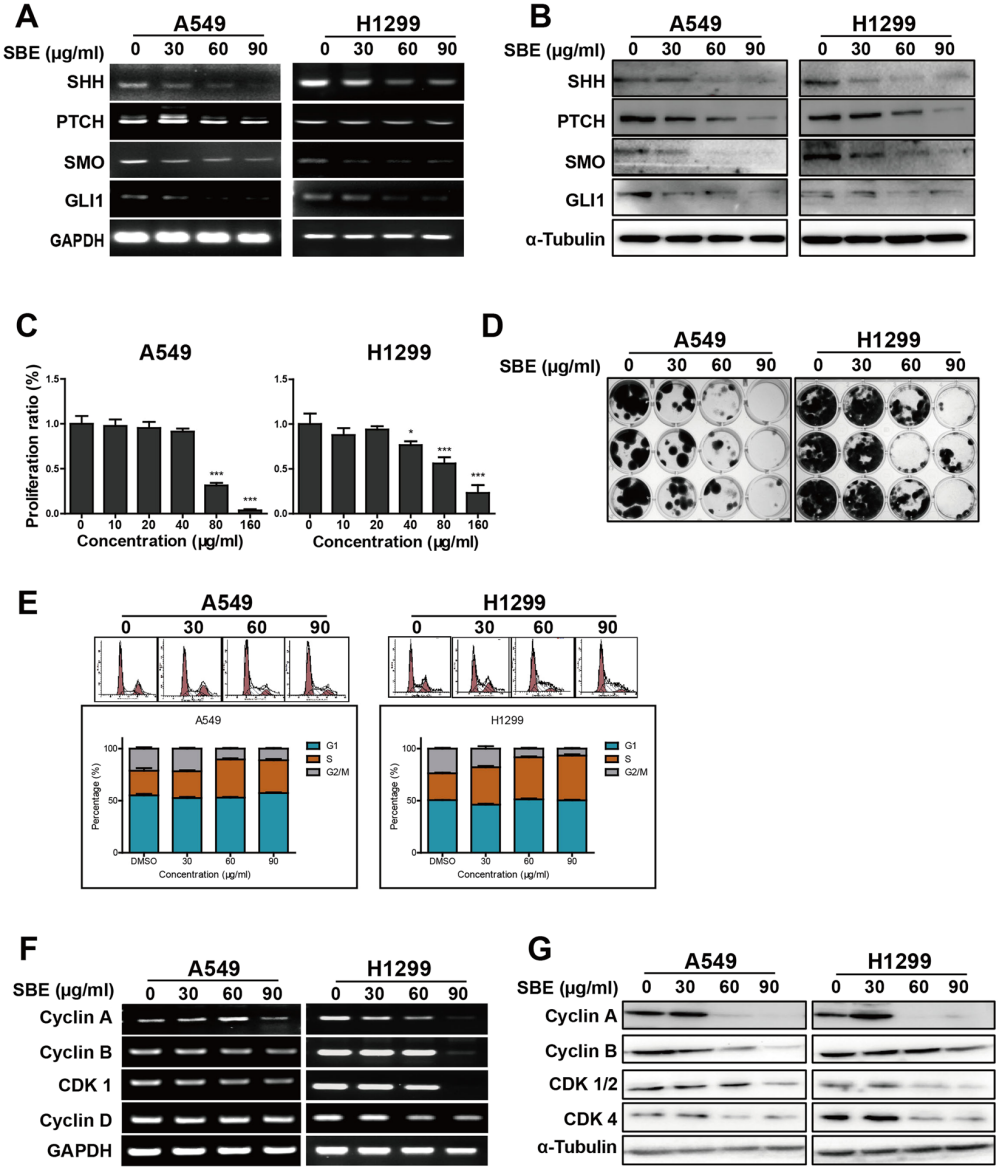
SBE inhibits cell proliferation and clonogenicity of NSCLC cells via selectively repressing SHH pathway and cell cycle checkpoint enzymes. (**A**) RT-PCR analysis demonstrating that SBE at 30, 60 and 90 μg/ml significantly decreased transcriptional activities of SHH pathway components (SHH, PTCH, SMO and GLI1) in A549 and H1299 cells. (**B**) Immunoblotting analysis demonstrating that SBE reduced protein level of SHH pathway components (SHH, PTCH, SMO and GLI1) in A549 and H1299 cells. (**C**) CCK-8 assay showing that SBE repress NSCLC cell proliferation in dose-dependent manner in A549(≥80 μg/ml) and H1299 (≥40 µg/ml) cells tested by CCK-8. (**D**) SBE repressed clonal formation of cells in dose-dependent manner in A549 and H1299 cells from 30 to 90 μg/ml. (**E**) Flowcytometry analysis demonstrating that SBE significantly induced G1/S phase arrest in A549 at 60 µg/ml or higher and H1299 cells at 30 µg/ml or more. Data were analyzed by ModFit software and presented as mean ± SD. (**F**) RT-PCR analysis demonstrating that SBE significantly inhibited transcriptional activities of cell cycle regulators (Cyclin A, Cyclin B, CDK1 and CDK4) in A549 and H1299 cells at 60 or 90 µg/ml concentration. (**G**) Immunoblotting analysis demonstrating that SBE decreased protein level of cell cycle regulators (Cyclin A, Cyclin B, CDK1 and CDK4) in A549 and H1299 cells at 60 or 90 µg/ml concentration.

SBE, as a traditional Chinese herb with especially high-effective and low-toxic efficacy in human breast and colon cancer via targeting SHH pathway to repress tumor angiogenesis^27, 30, 31^, was examined in its *in vitro* suppression of NSCLC progression and those potential pathways involved. RT-PCR analysis revealed that SBE at 30, 60 and 90 μg/ml significantly decreased transcriptional activities of SHH pathway components (SHH, PTCH, SMO and GLI1) at both mRNA and protein levels in A549 and H1299 cells (Fig. 3A and B). CCK-8 assay demonstrated that SBE repress NSCLC cell proliferation in dose-dependent manner in A549 (≥80 μg/ml) and H1299 (≥40 µg/ml) cells (Fig. 3C). As in Fig. 3D, SBE more significantly repressed clonal formation of cells in dose-dependent manner in A549 and H1299 cells from 30 to 90 μg/ml. Further flow cytometry analysis indicated that SBE significantly induced G1/S phase arrest in A549 at 60 µg/ml or higher and H1299 cells at 30 µg/ml or more (Fig. 3E), in which SBE significantly inhibited transcriptional and translational activities of cell cycle regulators (Cyclin A, Cyclin B, CDK1 and CDK4) in A549 and H1299 cells at 60 or 90 µg/ml concentration (Fig. 3F and G).

### SBE sensitizes DDP-induced tumor growth inhibition and distant-organ metastasis suppression of NSCLC *in vitro* and *in vivo*

**Figure 4:**
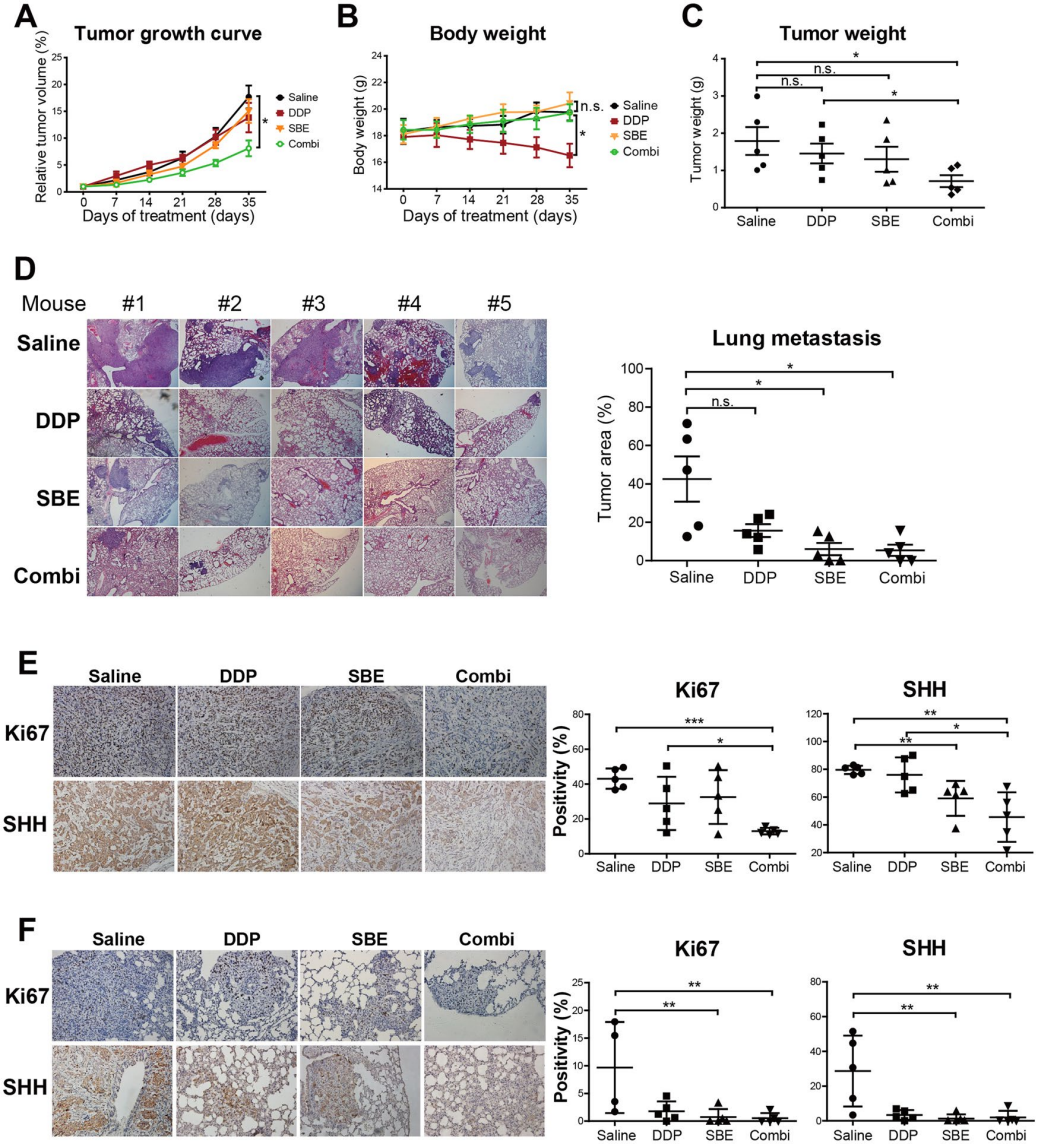
SBE alone or combined with DDP inhibits tumor growth and distant-organ metastasis of NSCLC *in vivo*. (**A**) Growth of A549 subcutaneous xenografts in nude mice. Treatment of SBE plus DDP significantly retard xenograft growth *in vivo* compared to Control (saline treated), SBE or DDP treatment alone groups. Relative tumor volume was calculated by normalizing actual tumor volume with corresponding tumor volume before treatment. (**B**) Body weights of nude mice bearing A549 xenografts exposed to SBE and/or DDP treatments demonstrating that SBE treatment alone did not affect the body weight of animals and its co-treatment with DDP arrested DDP treatment-induced body weight loss significantly. (**C**) Tumor weight from A549 xenografts. SBE plus DDP treatment more dramatically reduced tumor xenograft masses than SBE or DDP treatment alone. (**D**) Representative microscopic images (H&E staining) of lung metastasis tumor nodules from primary A549 subcutaneous xenografts in nude mice. Quantitation of total microscopic pulmonary tumor areas normalized to total lung tissue section areas showing that SBE or combined with DDP significantly reduced lung metastasis rate and DDP alone treatment did not inhibited lung metastasis significantly. HE staining photos were taken at 40 × and one picture from each mouse is shown as representative. Tumor area percentage was measured and calculated with image J software. (**E**) Immunohistochemistry staining of Ki67 (upper) and SHH (lower) in xenograft tumor tissues from different treatment group showing that SBE plus DDP more significantly repressed Ki67 and SHH expression than SBE or DDP alone. (**F**) Immunohistochemistry staining of Ki67 (upper) and SHH (lower) in lung tissues from different treatment group demonstrating that SBE plus DDP more significantly repressed Ki67 and SHH expression than SBE or DDP alone. Positivity was normalized with lung metastasis ratio in corresponding mouse. Representative pictures were taken at 200×. *P < 0.05, **P < 0.01. All the co-treatments of DDP and SBE were labeled as “Combi” in this figure.

While SBE alone significantly inhibits in cell growth and clonogenicity of NSCLC, it is also important to further interrogate whether SBE sensitizes NSCLC cells to chemotherapeutic agent DDP both *in vitro* and *in vivo*. Our *in vitro* wound healing studies (Fig. S1) demonstrated that SBE at low dose (40 µg/ml) mildly arrested the motility of A549 and H1299 cells and DDP at low dose (2 µg/ml) has no effects. However, co-treatment of SBE (40 µg/ml) with DDP (2 µg/ml) significantly and synergistically blocked those cell migrations. In the following *in vivo* analysis of inhibitory effects of SBE alone, or combined with DDP on NSCLC growth and metastasis tendency, we confirmed that treatment of SBE (50 mg/kg) with DDP (1.5 mg/kg) more significantly retarded xenograft growth than SBE or DDP treatment alone, in which there were no obvious body weight loss of animals during the whole treatments except DDP-only treatment group (Fig. 4A,B and C, Fig. S2A and B). Pathological examination of lungs from nude mice at the end of experiments identified the significant differences in lung metastasis from A549 xenografts of nude mice. Lung metastatic events occurred in 5 of 5 (100%) nude mice in control group, in which 3 of 5 mice have extensive large area metastasis in lung and the remaining two mice were affected by uneven wide distribution of focal metastasis. SBE alone robustly rescued four out of five mice from lung metastasis, leaving only one mouse with a few focal metastasis cancer cell islands without significant improvement in combined treatment with DDP (Fig. 4D). While mice treated with DDP alone acquired diffusive small and focal metastatic lesions rather than large metastatic tumor bulk as in the control group, the metastasis reduction resulted from DDP treatment was not statistically significant (Fig. 4D). For validation of SBE-induced *in vivo* repression of SHH pathway in NSCLC, Ki67 and SHH as proliferative and Hedgehog pathway marker respectively were analyzed in immunohistochemistry (IHC) staining of those xenografts and animal lungs with metastasis burdens (Fig. 4E and F, Fig. S3). Semi-quantitative IHC expression analysis of both Ki67 and SHH in xenografts revealed that SBE combined with DDP significantly downregulated expression of Ki67 and SHH compared to the control group and DDP treatment alone in which SBE alone was enough to downregulate the SHH activities relative to control group. (Fig. 4E). Similar IHC assay in lung metastatic lesions indicated that SBE alone or combined with DDP more dramatically reduced staining intensities of Ki67 and SHH compared to the control group (Fig. 4F).

### Downregulation of SMO magnifies inhibitory effects of SBE on NSCLC cell proliferation

**Figure 5:**
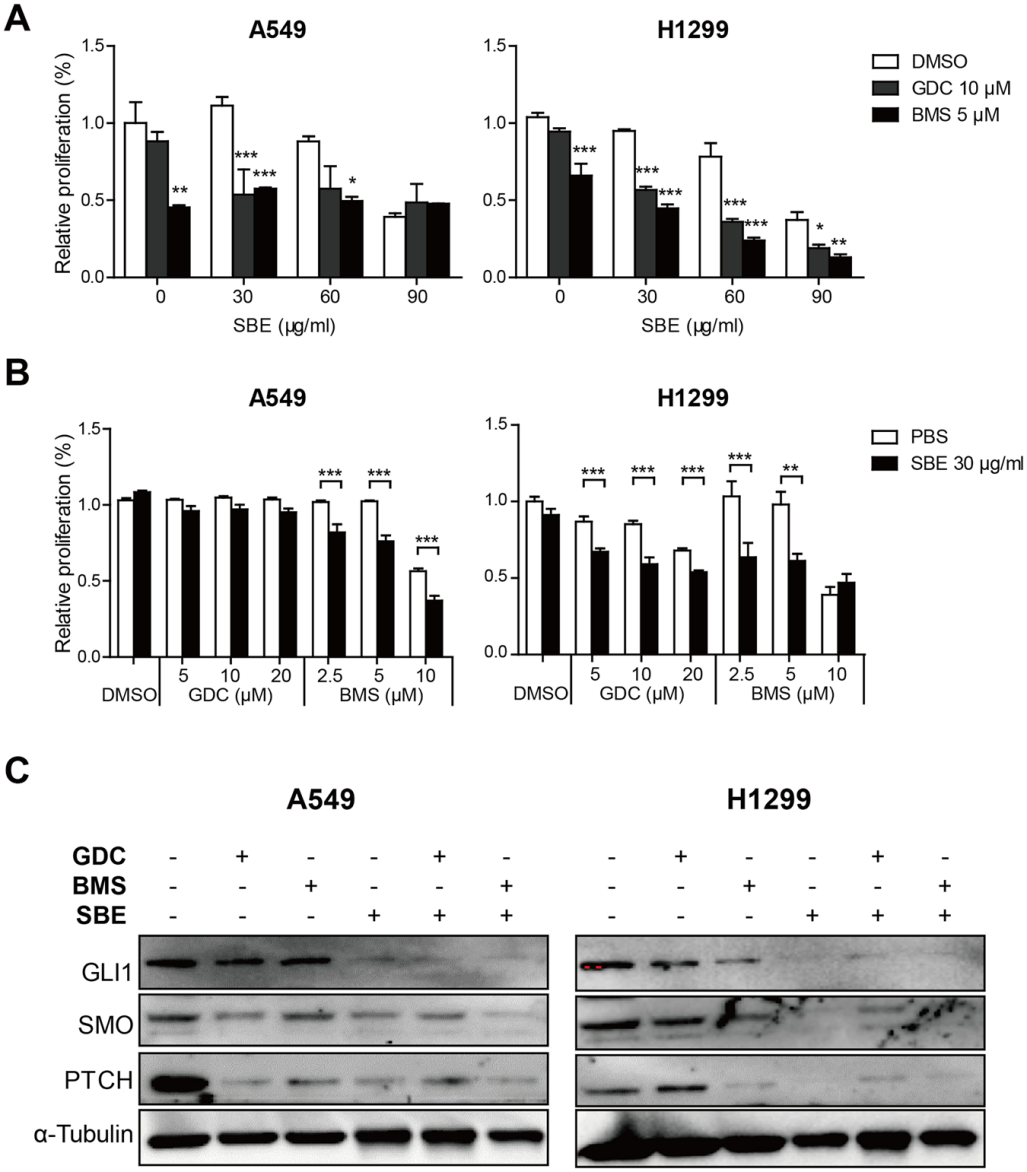
Downregulation of SMO magnifies inhibitory effects of SBE on NSCLC cell proliferation. (**A**) Proliferation assay indicating that GDC or BMS and various dose of SBE cooperatively repress NSCLC cell proliferation. (**B**) Proliferation assay showing that low dose of SBE sensitized anti-proliferative effects of SMO inhibitor (GDC and BMS) on A549 and H1299 cells. (**C**) Immunoblotting analysis demonstrating that SBE enhanced GDC or BMS-induced repression of GLI1, SMO, and PTCH on protein level.

Given that the viability of A549 and H1299 was slightly affected with exposure to SMO inhibitors GDC and BMS alone, here we tested whether any potential additive or even synergistic effects of SBE with one of those two compounds on tumor cell proliferation were experimentally significant. We found that GDC treatment at 10 μM significantly enhanced SBE-induced growth inhibition at 30 μg/ml or more, but BMS did not in A549 cells (Fig. 5A). For H1299 cells, both GDC (10 μM) and BMS (5 μM) consistently sensitized SBE-induced cancer cell proliferation arrest at 30 μg/ml or higher, while SBE alone at 30 μg/ml have no effects on A549 cells and only marginal effects on H1299 cells (Fig. 5A). To further validate the combinatory effect between SBE and SMO inhibitors, we then fixed SBE at low dose (30 µg/ml) and combine with various dose of GDC or BMS. As indicated in Fig. 5B, addition of SBE remarkably enhanced inhibitory effect of BMS at 2.5 and 5 µM in both cell lines while improve GDC at 10 and 20 µM in H1299 cells. Subsquent immunoblotting analysis demonstrated that SBE systemically inhibited SHH pathway and aggravated GDC or BMS-induced repression of GLI1, SMO, and PTCH in protein level in A549 and H1299 cells (Fig. 5C).

### Up-regulation of SMO resists the SBE-induced reduction in invasive growth of NSCLC cells

**Figure 6:**
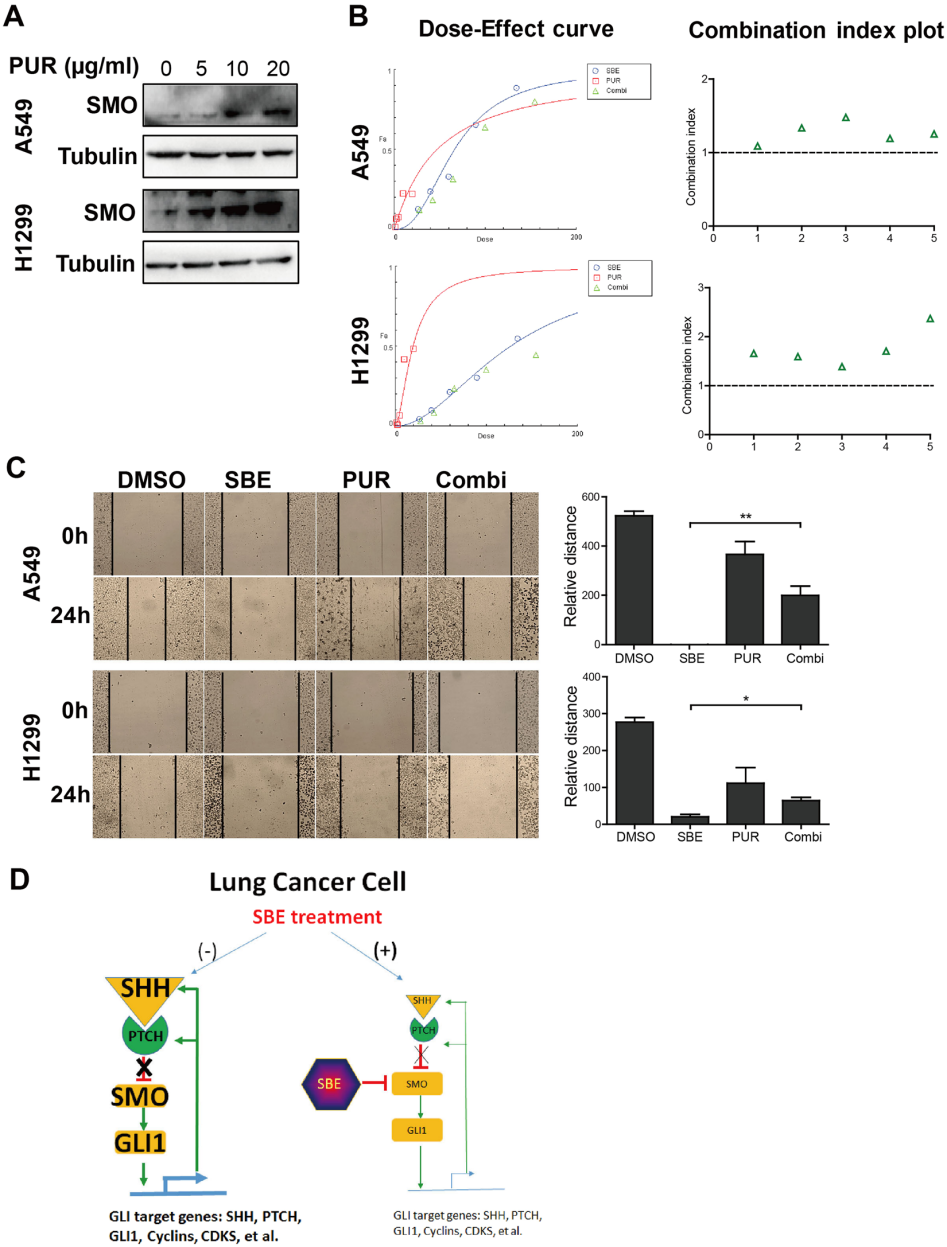
Up-regulation of SMO resists SBE-induced reduction in invasive growth of NSCLC cells. (**A**) Immunoblotting analysis illustrating that SMO agonist Purmorphamine (PUR) activated SMO expression in A549 and H1299 cells. (**B**) CompuSyn Software-assisted analysis of Dose-Effect curve (left) and Combination Index Plot (right) for SBE, PUR, or both showing. SBE was analyzed at dose from 26.7 to 135 µg/ml and PUR was tested at dose from 1.25 to 20 µM. (**C**) Wound healing assay showing that PUR antagonized SBE-induced repression in invasive growth of A549 and H1299 cells. (**D**) Schematic diagram depicting the SBE-mediated selective targeting inhibition of SHH-cell cycle signal axis in lung cancer therapy. As indicated in this diagram, SBE directly and selectively targets SMO both transcriptionally and translationally causing significant inhibition of GLI1 and its downstream targets including SHH signaling components and cell cycle regulators. All the co-treatments of SBE and PUR were labeled as “Combi” in this figure.

To test the specific targeting of SMO by SBE, SMO agonist Purmorphamine (PUR) was introduced in A549 and H1299 cells for examining its roles in antagonizing SBE-induced cell invasive growth. First, PUR is a strong and specific agonist for SMO which dramatically and dose-dependently activated the expression of SMO in A549 and H1299 cells as in Fig. 6A. For drug interaction assay, according to Chou’s method^32^, the drugs are considered to have synergistic, additive or antagonistic effect with each other when combination index below, equal or above 1.0^32^. The following CompuSyn Software-assisted analysis of Dose-Effect curve (Fig. 6B, left) and Combination Index Plot (Fig. 6B, right) in A549 and H1299 cells treated with SBE, PUR, or both revealed that combination index for co-treatment of both SBE and PUR are always higher than 1.1, which supports the direct and selective antagonizing relationship between SBE and PUR based on these *in vitro* physiological functions analysis. Wound healing assay revealed that PUR, despite of inducing cytotoxicity when applied alone, counteracted high dose SBE (160 µg/ml) -induced metastasis inhibition (Fig. 6C). Our data strongly indicate that the SBE selectively and significantly targets SMO, the key regulator of SHH signaling, to repress the intensity of entire SHH pathway for inhibition of NSCLC progression and chemo-resistance developed from DPP treatment clinically.

Collectively, all the data above suggest that SBE-mediated selective targeting inhibition of SHH-cell cycle signal axis in lung cancer therapy is achieved via its direct and selective inhibition of SMO at both transcriptional and translational levels which consequently leads to significant inhibition of GLI1 and its downstream targets including SHH signaling components and cell cycle regulators (Fig. 6D).

## Discussion

In this study, we tested the tumoricidal activity of SBE in human lung cancer and identified the critical druggable targets for SBE in the deregulated molecular signaling wiring involved in lung carcinogenesis. We validated efficacy of SBE in repressing SHH signaling pathway to block NSCLC progression and confirmed that SBE selectively induced repression of aberrantly activated SHH signaling pathway leading to disruption of the SHH-mediated non-small cell lung carcinoma progression and metastasis via cell cycle arrest. We also found that SBE significantly sensitized lung cancer cell to chemotherapeutic agent DDP via repressing SHH components *in vitro* and *in vivo*. Our results suggest that SBE is a promising therapeutic drug candidate for NSCLC which specifically and sensitively target SHH signal pathway.

Limited improvements in more effective treatments and prevention of lung cancer still keep it as the most aggressive malignant tumor with one of the lowest survival rates and leave most patients with this type of cancer present advanced and unresectable disease without choices but palliative treatment such as chemotherapy and radiotherapy. Moreover, some normal tissues and cells are destroyed as well by this method of treatment. Therefore, searching for novel natural compounds with low toxicity and high selectivity of killing cancer cells is demanding in cancer therapy.

One of Traditional Chinese medicines (TCMs), *Scutellariabarbata D. Don* extraction (SBE) have been being applied independently or combined with modern medicine to improve symptoms, enhance quality of life, prevent recurrence and metastasis, and prolong survival in cancer patients in China for many years^21, 22, 25, 33, 34^. In our studies, SBE significantly inhibited the *in vitro* growth of human non-small cell lung cancer A549 and H1299 cells in dose-dependent manners. Western blot analysis indicated that SBE dose-dependently down-regulated the expression of SHH signal cascade members, eventually leading to the cell cycle arrests in lung cancer cells. Moreover, SBE significantly suppressed the lung metastatic colonization of A549 cell from xenografts *in vivo*. Our findings suggest that SBE may have the wide therapeutic and/or adjuvant therapeutic application in the treatment of human lung cancer.

Key regulators of Hedgehog pathway, particularly SHH, SMO and GLI1 are aberrantly stimulated in various cancers including lung cancer^8, 12–14^ and have crosstalk with other neoplastic related cascade like RAS/RAF/MEK/ERK, PI3K/AKT/mTOR, EGFR, and Notch^9, 35^. Deregulated Sonic Hedgehog (SHH) pathway facilitates the initiation, progression and metastasis of NSCLC, confers drug resistance against targeted therapy and renders a lung cancer therapeutic interference option to cancer patients with poor prognosis^36–38^. Substantial studies have proved close relationship between aberrantly activated Hedgehog cascaded and chemo-drug resistance in lung cancer while downregulation of SHH pathway resensitizes cancer cells to conventional chemotherapy^7, 18, 20, 39, 40^. SHH-specific chemotherapeutic drugs are not clinically available yet. SBE is reported recently to repress tumor angiogenesis via suppression of Hedgehog signaling and to enhance G1/S arrest in human colon carcinoma cells by modulating a number of signaling pathways associated with the cell cycle^26, 27^. However, the underlying mechanisms governing the antitumor role of SBE in lung cancer and cancer metastasis are still not fully explored. To further elucidate the mechanism underlying the tumoricidal activity of SBE in this study, we screened and evaluated the specificity of SBE in repressing SHH signaling pathway to block NSCLC progression. Our study revealed that SHH signal pathways were aberrantly activated in lung cancer tissues and cells which conferred more proliferative and invasive phenotypes to human lung cancer cells and leading to adverse prognosis in patients (Fig. 1). We confirmed that SBE specifically repressed SHH signaling pathway to interfere the SHH-mediated non-small cell lung carcinoma progression and metastasis via arresting cell cycle. We also found SBE significantly sensitized lung cancer cell to chemotherapeutic agent DDP via repressing SHH components *in vitro* and *in vivo*. Mechanistic investigations revealed that SBE transcriptionally downregulated SHH signaling pathway SMO, PTCH, GLI1, which interacted with cell cycle checkpoint enzymes to arrest control cell cycle leading to cellular growth and migration blockade. Our results suggest that SBE is a promising therapeutic drug candidate for NSCLC which specifically and sensitively target SHH signal pathway.

Given the SBE-induced pervasive inhibition of SHH signaling especially including PTCH which is supposed to repress SMO in normal mammalian cells, PTCH in lung cancer cells is still functioning as a repressor of hedgehog cascade reaction chain and is overwhelmingly suppressed or neutralized by overexpressed SHH. We applied a model as indicated in Fig. 6D to further define SBE-mediated selective targeting inhibition of SHH-cell cycle signal axis in lung cancer therapy. In lung cancer cells, SHH is overexpressed and competitively binds PTCH leading to its dysfunctions in arresting SMO activation followed by upregulation of GLI1 which consequently induces the transcription activities of GLI1 target genes including Hedgehog cascade members (SHH, PTCH, and GLI1) and cell cycle components (Cyclins and CDKs). Treatment of SBE directly and selectively represses SMO both transcriptionally and translationally causing significant inhibition of GLI1 and its downstream targets. Meanwhile, reduction in transcription of Hedgehog cascade members as downstream targets of GLI1 back forwardly further attenuates the SHH signaling in lung cancer cells. Alteration in PTCH expression does not affect oncogenic functions of Hedgehog cascade driven by SHH, SMO, and GLI1 in lung cancer cells. SBE indirectly induces cell cycle arrests via repressing cyclins and CDKs in this model.

Studies for SBE are promising. Although we have achieved significant results, there are still many issues that need to be explored in in-depth studies, such as the specificity in SBE-induced repression of SHH to enhance chemotherapeutic treatment of lung carcinoma drug resistance. In our study, we only found the SBE sensitized lung cancer cell to chemotherapeutic agent DDP, the drug-resistance interference by SBE are not fully investigated, and the underlying mechanism needs to be further explored through in-depth studies. In addition, several studies demonstrated that activation of SHH cascade is highly related to resistance to EGFR inhibitors in NSCLC^36–38^, thus whether SBE facilitates EGFR inhibitor therapy by repressing SHH pathway activity is of great interest to us.

In summary, SBE can function as an efficient non-resistance therapeutic drug for the treatment of lung cancer. SBE selectively represses the expression of SHH and arrests cell cycle progression to retard the cell proliferation and invasive or metastatic colonization of NSCLC cells. Our results suggest the potential for SBE as a chemotherapeutic agent for repressing tumorigenesis and progression of NSCLC via targeting SHH signaling in human non-small cell lung cancer. Inhibition of cell growth induced by SBE is associated with disturbance of cell cycle progression of the lung cancer cells. SBE induces substantial growth and metastasis inhibition of lung cancer cells *in vitro* and *in vivo* which grants any further clinical investigation.

## Materials and Methods

### Cell lines and chemicals

The non-small cell lung cancer cell lines A549 and H1299 were obtained from American Type Culture Collection (ATCC; Manassas, VA), and maintained in RPMI medium supplemented with 10% fetal bovine serum (Hyclone, USA) and 1% penicillin/streptomycin. Cells were incubated at 37 °C with 5% CO2. There are genetic differences between A549 and H1299 cell especially in p53 status per the published literature. A549 cells have wild type P53 and intact function to exert apoptosis and H1299 cells have a homozygous partial deletion of the TP53 gene and as a result, do not express the tumor suppressor p53 protein which in part accounts for their proliferative propensity^3^. All cell lines were routinely tested using a mycoplasma-contamination kit (R&D). Informed consent was obtained under a protocol reviewed and approved by the Institutional Review Board at the Binzhou Medical University Committee. GDC-0449 (S1082), BMS-833923 (S7138) and Purmorphamine (S3042) were purchased from Selleck Chemicals. Doses of chemicals are indicated for particular experiment.

### Human tissues

Five pairs of human lung cancer and normal lung specimens were collected in Affiliated Hospital of Binzhou Medical University with written consents of patients and the approval from the Institute Research Ethics Committee at the Binzhou Medical University. Primary human samples were processed and analyzed with written consents of patients at the Binzhou Medical University under a protocol reviewed and approved by the Institutional Review Board at the Binzhou Medical University. All tissues were immediately snap-frozen with a portion of harvested tissue sent for immediate histologic confirmation by an independent, anatomic pathologist in a blinded manner. All these five human lung cancer samples with paired pathologically normal lungs were used for RT-PCR and immunoblot analysis.

### Analysis of publicly available datasets

To analyze correlation between SMO or GLI1 expression level and prognostic outcome of patients, Kaplan-Meier survival curves of NSCLC patients with low and high expression of SMO, GLI1 or SHH were generated using Kaplan-Meier Plotter (www.kmplot.com/analysis)^29, 41^.

### SBE extraction and purification

Dried and powdered whole plants of S. barbata (5.0 kg) were extracted using MeOH (3 × 10 liters, 7 days each time) at room temperature and then concentrated *in vacuo* to obtain the crude extract. The crude extract was dissolved in DMSO for storage.

### Cell proliferation assay

Cells were seeded at density of 2000 cells/well into 96-well plate one day before drug treatment. Cell counting kit-8 (CCK-8, Dojindo, CK04) test was performed 48 hours after various drug incubation according to manufacturer’s guide. The absorption and reference wavelength was measured separately at 450 nm and 630 nm.

### Cell cycle analysis

Cells were collected and washed with pre-cold PBS. For 2 × 10^5^ cells resuspended with 400 μl propidium iodide (PI) mixture (including 200 μg/ml PI, 100 μg/ml RNase, 0.2%Triton X-100) and incubated at 4 °C for 30 min. Fluorescent signals were measured at FL-2 channel with CFlow Plus package from Accuri C6 and analyzed with ModFit software.

### Would healing assay

Cells were seeded into 12-well plate at 10^5^ cells/well one day before treatment. On the second day, upon 90% confluence, cells were scraped across using a plastic 200 µl tip and washed twice with PBS followed by incubation with serum free medium supplemented with corresponding drugs for 24 hours. Photomicrographs were taken with Olympus light microscope of a same area at before and 24 hours after treatment. Relative distance of the remaining wound area was normalized to the initial wound area and quantified with ImageJ software package. The relative distance shown in all our wound healing studies is calculated as follows: Relative distance = Wound width at the beginning of experiment -Wound width at the end of experiment for 24 hours. Experiments were performed in triplicates.

### Clonal formation assay

Cells were seeded into 12-well with 300 cells per well and cultured for 24 hours followed by being exposed to drugs for another 24 hours. Cells were then cultured in drug-free medium for another 10 to 15 days until clones of around 50 cells were formed. Cell clones were fixed with 4% paraformaldehyde for 0.5 hour and stained with crystal violet (Sangon) for 15 min before optical imaging.

### RT-PCR

RNA was extracted with Trizol (Sangon) and reverse transcribed into cDNA with RevertAid First Strand cDNA Synthesis Kit (Fermentas) according to manufacturer’s instructions. Polymerase chain reactions were performed with 2 × EasyTaq® PCR SuperMix (TransGen Biotech) according to manufacturer’s instructions. All primer sequences were included in supplemental Table 1.

### Immunoblotting analysis

Whole-cell protein lysates were separated on sodium dodecyl sulfate-polyacrylamide gels (SDS-PAGE) and transferred to polyvinylidene difluoride (PVDF) membranes (Millipore) by electroblotting. Membranes were incubated with the respective primary antibody at 4 °C overnight, followed by horseradish peroxidase (HRP)-conjugated secondary antibodies and finally detected via an enhanced chemiluminescence signal (ECL, Amersham). Photometric analyses of immunoblots were carried out using the Image Lab software package. Antibody information is available in supplemental Table 2.

### Transfections

The cells were transiently transfected in 6-well plate using Lipofectamine 2000 (Invitrogen) according to the manufacturer’s protocol. Chemically synthesized double-stranded siRNAs for Gli1 (Santa Cruz, sc-37911) and non-target siRNA as control were transfected in parallel at a concentration of 50 nmol into the cells. Immunoblot and colonal formation assay were performed 48 hours post transfection.

### *In vivo* experiment

3- to 5-week old female balb/c athymic (nu/nu) mice were purchased from Charles River Laboratories in Beijing. All mice were housed in a biosafety level 2 lab and maintained in accordance with institutional guidelines at Binzhou Medical University. All animal experiments were performed with the approval of Binzhou Medical University Committee on Animal Care. Mice were subcutaneously injected with 5 × 10^6^ A549 cells and randomly divided into different groups (5 mice per group) when the diameter of established tumors reached approximately 5 mm. Xenografted mice received vehicle (200 μl saline), DDP (1.5 mg/kg), SBE (50 mg/kg), or their combination intraperitoneally three times a week. Body weight and tumor volume was measured every second day. Tumor volume was calculated as 0.5 × a^2^ × b (a and b represent tumors short and long diameter respectively). Mice were euthanized and executed after 5 weeks’ treatment and tumors were measured. Tumor tissues were collected from xenografts at the end of treatment and analyzed by immunohistochemistry staining.

### Immunohistochemical analysis

Mice tumor and lung tissues were fixed in 4% paraformaldehyde in phosphate-buffered saline (PBS) overnight and subsequently embedded in paraffin wax. Sections were cut at a 4 μm and stained with hematoxylin andeosin (HE) for histological analysis. Immunohistochemical analysis were performed with standard protocol. Sections were deparaffinized followed by antigen retrieval by heating for 15 min in EDTA buffer (pH 9.0) in microoven. After treated with 3% hydrogen peroxide, tissues were incubated with primary antibodies or PBS as negative control at 1: 250 dilutions with 5% BSA at 4 °C overnight followed by corresponding Hydrogen-peroxide oxidoreductase (HRP) conjugated secondary antibodies diluted at 1:200 by 5% BSA at 37 °C for 1 hour. The Diaminobenzidine (DAB) Detection System (Solarbio, China) was used for visualization according to manufacturer’s instructions. Digital images were collected by image-processing software (Olympus) and histologic evaluation of tumor and lung sections was performed by experienced pathologist. At least ten fields were randomly taken from each slide and average positivity from each mouse was calculated as the mean positivity from all fields.

### Statistical analysis

Data were analyzed with Graphpad Prism 5 software and results were presented as the means ± SD. The two-tail Student’s t-test was used to compare two groups and ANOVA withTukey post-hoc test was used to compare multiple groups. A *P*-value < 0.05 was considered statistically significant and marked by an asterisk. A *P*-value < 0.01 was marked by two asterisks. A *P*-value < 0.001 was marked by three asterisks.

## Electronic supplementary material

Supplementary figures and tables
