# Author Correction: Disruption of SHH signaling cascade by SBE attenuates lung cancer progression and sensitizes DDP treatment

**DOI:** 10.1038/s41598-020-74978-x

**Published:** 2020-10-22

**Authors:** Jing Du, Weiwei Chen, Lijuan Yang, Juanjuan Dai, Jiwei Guo, Yan Wu, Kaikai Gong, Jian Zhang, Ning Yu, Zhen Xie, Sichuan Xi

**Affiliations:** 1 Cancer Research Institute, Binzhou Medical University Hospital, Binzhou, 256600 People’s Republic of China; 2 Department of Pathology, Binzhou City People’s Hospital, Binzhou, 256610 People’s Republic of China; 3 Department of Pathology, Binzhou Medical University Hospital, Binzhou, 256600 People’s Republic of China; 4 Department of Thoracic Surgery, Binzhou Medical University Hospital, Binzhou, 256600 People’s Republic of China

Correction to: *Scientific Reports* 10.1038/s41598-017-02063-x, published online 15 May 2017

This Article contains errors.

In Figure 6C, the image for SBE at 0h (A549 cells) was inadvertently duplicated for Combi at 0 h (A549 cells). The correct Figure 6C appears below as Fig. 1.

**Figure 1:**
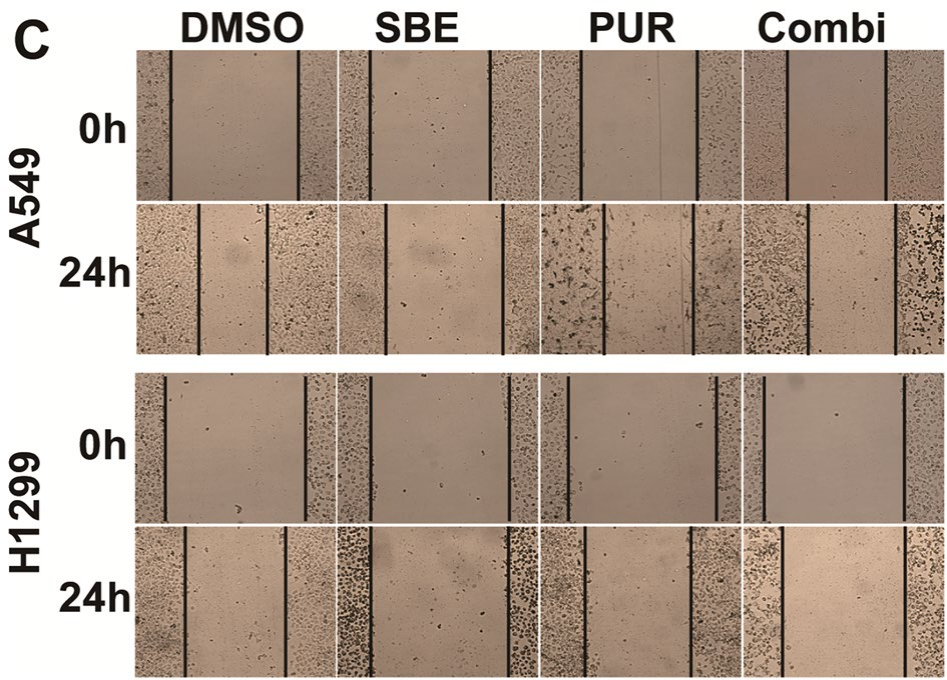
A corrected version of Figure 6C in the Article.

In Figure 3F, “CDK4” was mislabelled as “Cyclin D”. The correct Figure 3F appears below as Fig. 2.

**Figure 2:**
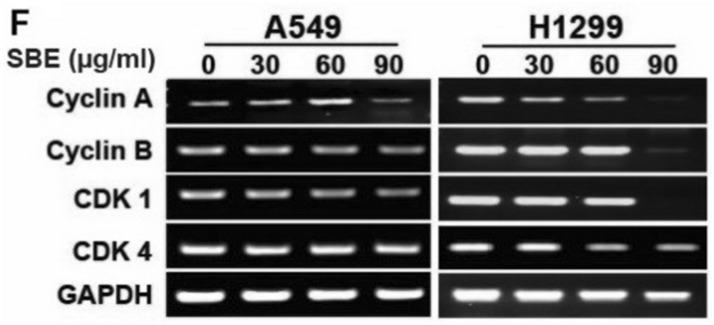
A corrected version of Figure 3F in the Article.

These changes do not affect the conclusions of the Article.

