# Supplementary material for: Disruption of SHH signaling cascade by SBE attenuates lung cancer progression and sensitizes DDP treatment
Source: Sci Rep. 2017 May 15;7:1899. doi: 10.1038/s41598-017-02063-x (PMC5432500; doi:10.1038/s41598-017-02063-x)
Supplement: Supplementary file 1 — Supplementary figures and tables [file 41598_2017_2063_MOESM1_ESM.pdf]

Disruption of SHH signaling cascade by SBE attenuates lung cancer progression and sensitizes DDP treatment

Jing Du,[1#] Weiwei Chen,[1#] Lijuan Yang,[1#] Juanjuan Dai,[1] Jiwei Guo,[1] Yan Wu,[1] Kaikai Gong,[1] Jian Zhang,[2]Ning Yu,[3] Zhen Xie[4] and Sichuan Xi[1*]

[1]Center Research Institute, Binzhou Medical University hospital, 256600 Binzhou, P.R. China;

[2]Department of Pathology, Binzhou City People's hospital, 256610 Binzhou, P.R. China;

[3]Department of Pathology, Binzhou Medical University hospital, 256600 Binzhou, P.R. China;

[4]Department of thoracic surgery, Binzhou Medical University hospital, 256600 Binzhou, P.R. China

[#]These three authors contributed equally to this work.

[*]Corresponding author:

Sichuan Xi, MD, PhD

Cancer Research Institute, Binzhou Medical University Hospital, Binzhou 256603, P.R. China ; Tel: +86 05433258276; FAX: +86 05433258276;

Email: xh_xi@yahoo.com

Running Title: SBE inhibits SHH signaling in lung cancer

Keywords: NSCLC, SBE, SHH signaling pathway, Cell cycle arrest and DDP sensitization

Figure S1

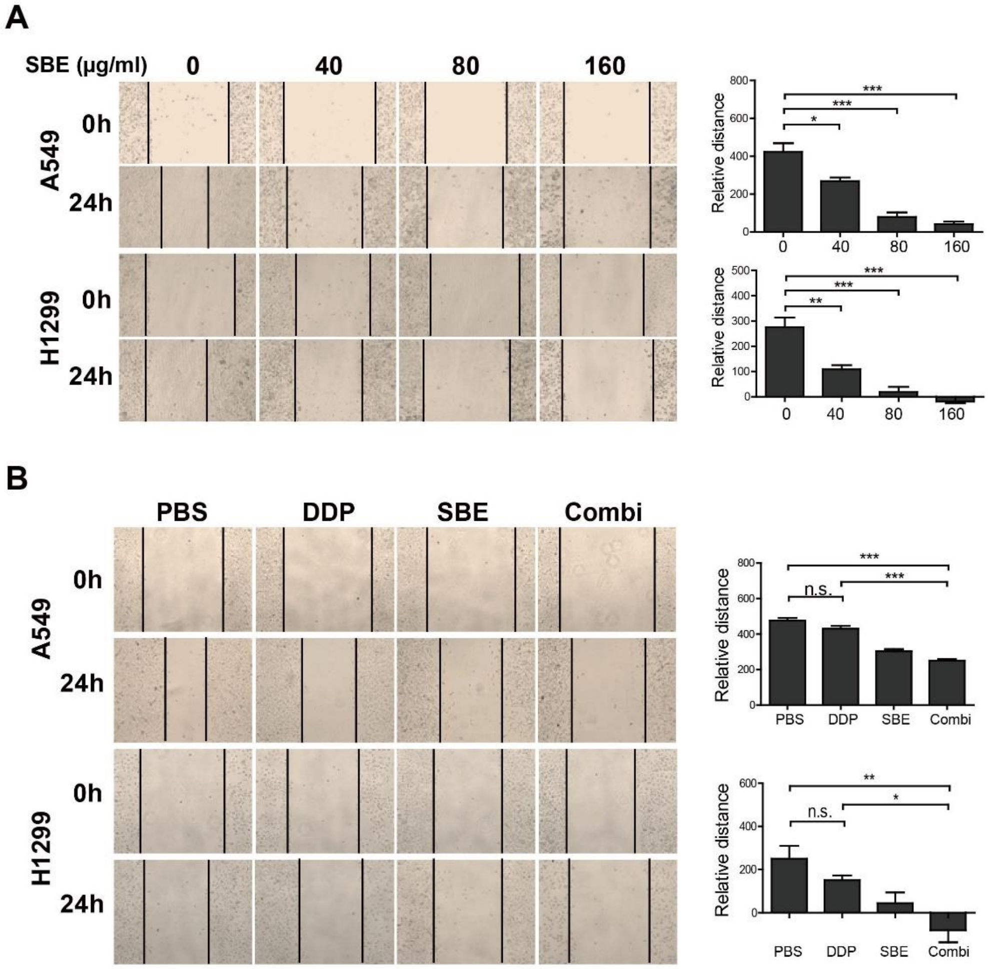

Figure S1. SBE sensitizes DDP-induced suppression of invasive growth in NSCLC cells in vitro

(A) Wound healing assay demonstrating that treatment of SBE for 24 hours inhibited cell migration of A549 and H1299 in dose-dependent manner (from 0 to 160 μg/ml).

(B) Further quantitative analysis of scar scratch test showing that SBE at low dose (40μg/ml) mildly arrested the motility of A549 and H1299 cells and DDP at low dose (2μg/ml) has no effects. However, Co-treatment of SBE (40μg/ml) with DDP (2μg/ml) significantly and synergistically blocked those cell migrations.

Figure S2

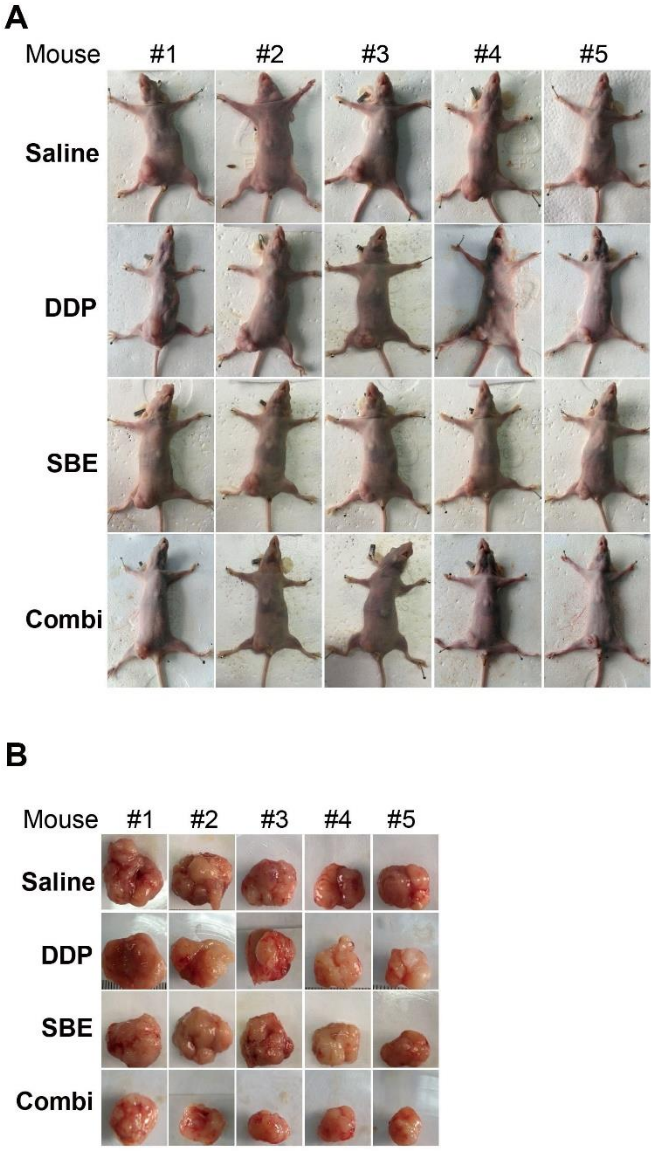

Figure S2. SBE interferes the SHH-mediated NSCLC progression in vivo

(A) Images for all the tumor bearing mice with various treatments. (B) Images for all the xenograft tumors from bearing mice with various therapy.

Figure S3

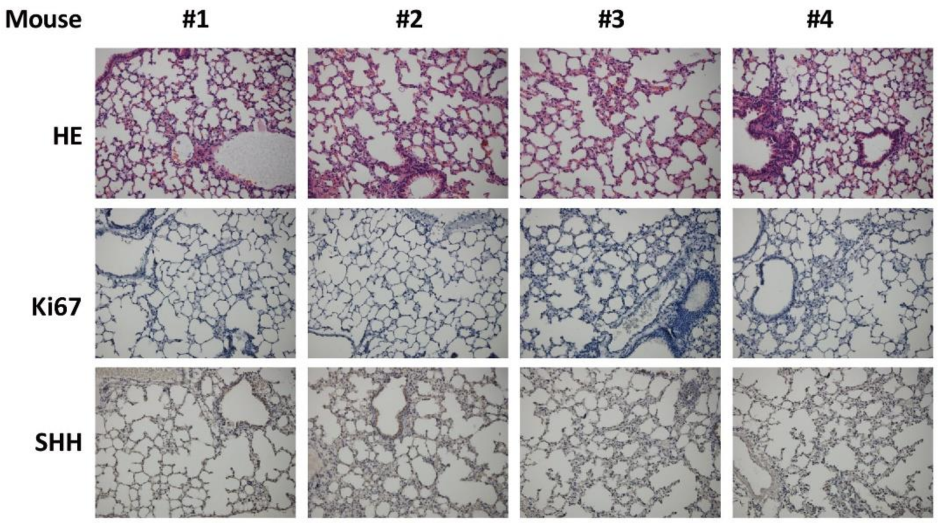

Figure S3. Negative controls for immunohistochemistry staining of Ki67 and SHH in Figure 4D/E/F. Representative immunohistochemistry staining of Ki67 and SHH and H&E staining from normal mouse lung tissues serve as negative controls.

Supplemental Table 1

Target gene	Sequence
SHH	For: TACTCGCAGCTGCTCTACCA
	Rev: TGTCTTTTTGCTTTGCGTTGC
PTCH	For: CAGCACTGGAAAACTCGTCA
	Rev: TCTGATGAACCACCTCCACA
SMO	For: GGGAGGCTACTTCCTCATCC
	Rev: GGCAGCTGAAGGTAATGAGC
GLI1	For: CCCAATCACAAGTCAGGTTCCT
	Rev: CCTATGTGAAGCCCTATTTGCC
Cyclin A	For: TGGAAAGCAAACAGTAAACAGCC
	Rev: GGGCATCTTCACGCTCTATTT
Cyclin B	For: CATGGTGCACTTTCCTCCTT
	Rev: AGGTAATGTTGTAGAGTTGGTGT
CDK 1	For:TGGATCTGAAGAAATACTTGGATTCTA
	Rev: CAATCCCCTGTAGGATTTGG
CDK 4	For: ACCAGATGGCACTTACACCC
	Rev: ACCAGATGGCACTTACACCC

Supplemental Table 2

Antibody Name	Dilution	Company
**Primary Antibody**		
Anti-Sonic Hedgehog antibody [EP1190Y] ab53281	1:1000	Abcam
Anti-Patched / PTCH1 antibody (ab53715)	1:1000	Abcam
Anti-Smoothened antibody ab72130	1:1000	Abcam
Anti-Gli1 antibody ab151796	1:1000	Abcam
α-Tubulin (11H10) Rabbit mAb	1:2000	Cell Signaling Technology
Anti-Ki67 antibody [SP6] ab16667	1:200	Abcam
Cdk1/Cdk2 (AN21.2): sc-53219	1:500	Santa Cruz
Cdk4 (C-22): sc-260	1:500	Santa Cruz
Cyclin A (W250) polyclonal antibody	1:500	Bioworld
Cyclin B (I120) polyclonal antibody	1:500	Bioworld
**Secondary Antibody**		
Anti-mouse HRP Linked Antibodies	1:1000	Cell Signaling Technology
Anti-rabbit HRP Linked Antibodies	1:1000	Cell Signaling Technology
